# Narcissistic Personality Disorder through psycholinguistic analysis and neuroscientific correlates

**DOI:** 10.3389/fnbeh.2024.1354258

**Published:** 2024-07-17

**Authors:** Dalia Elleuch

**Affiliations:** Faculty of Letters and Humanities, University of Sfax, Sfax, Tunisia

**Keywords:** Narcissistic Personality Disorder, psycholinguistic analysis, neuroscientific correlates, grandiosity, behavioral neuroscience

## 1 Introduction

According to the *Diagnostic and Statistical Manual of Mental Disorders, fifth edition* (DSM-5) by the American Psychiatric Association (APA), Narcissistic Personality Disorder (henceforth NPD) is a psychological condition marked by enduring traits of grandiosity, fantasies of boundless power, and an insatiable desire for admiration, including cognitive, affective, interpersonal, and behavioral aspects (American Psychiatric Association, 2013). It profoundly influences an individual's self-perception, interpersonal relationships, and psychosocial wellbeing. The complex web of narcissistic traits, ranging from an exaggerated sense of self-importance to a pervasive lack of empathy, poses significant challenges not only to those directly affected but also to the broader societal setting (di Giacomo et al., 2023). This study focuses on how language use in individuals with NPD reflects and reveals the core cognitive and emotional features of the disorder, particularly grandiosity, entitlement, and empathy deficits.

The term “Narcissism” originated in Greek mythology, specifically in the tale of Narcissus, a hunter who became fixated on his reflection (Cleveland Clinic, 2023). In contemporary psychology, NPD extends beyond a fixation on physical appearance, encompassing traits such as intelligence, charisma, success, and power (Kacel et al., 2017). This broader conceptualization forms the basis for the current exploration, acknowledging the complex nature of narcissism. Estimating the prevalence of NPD proves challenging due to its often covert manifestations, suggesting that between 0.5 and 5% of the population in the United States may experience NPD, with a higher occurrence among men (Rosenthal et al., 2020). However, the covert nature of narcissistic beliefs and behaviors complicates accurate assessments, highlighting the elusive nature of this condition. Hence, understanding the manifestations of NPD remains challenging, with experts pointing to a constellation of factors that contribute to its development (Weinberg and Ronningstam, 2022; Mitra, 2023). Research on NPD was conducted from various perspectives and disciplines, including genetics (Luo et al., 2014; Luo and Cai, 2018), observation and imitation (Obhi et al., 2014), negative childhood experiences (Clemens et al., 2022), parenting styles (van Schie et al., 2020), and cultural influences (Li and Benson, 2022).

Beyond its primary manifestations, NPD is a complex mental health condition often comorbid with other disorders, such as mood disorders, bipolar disorder, and substance use disorders (Mitra, 2023; Yaqoob and Ahsan, 2023). This comorbidity creates significant challenges in diagnosis and treatment. While traditional research focuses on the behavioral manifestations of NPD, a gap exists in understanding how language use reflects the core cognitive and emotional features of the disorder.

This study aims to address this gap by employing a multidisciplinary approach that integrates psycholinguistic analysis with established neuroscientific knowledge of NPD, potentially informing the development of clinical practices and patient-centered diagnostic and treatment approaches.

## 2 Methodology

This study employs a multidisciplinary approach integrating insights from psycholinguistics, clinical psychology, and neuroscience. The research design includes a thorough review of relevant literature and an exploration of neuroscientific findings. The foundation of this study rests on a review of academic literature, including peer-reviewed articles and clinical studies related to NPD to achieve the following purposes:

1. Defining NPD: A critical analysis of established diagnostic criteria outlined in the American Psychiatric Association's *Diagnostic and Statistical Manual of Mental Disorders: Fifth Edition Text Revision DSM-5-TR*™ (American Psychiatric Association, 2022), which forms the basis for understanding the core features of NPD.
2. Exploring psycholinguistic manifestations: Existing research on the psycholinguistic patterns and communicative behaviors associated with NPD is examined through a systematized review, delving into studies that analyze speech patterns, language use, and conversational dynamics of individuals with NPD. PubMed, PubMed Central, and PsycINFO databases are searched using keywords such as “Narcissistic Personality Disorder,” “speech patterns,” “language use,” and “conversational dynamics” to identify studies analyzing these aspects in individuals with NPD. The process involves a two-step screening. Initially, titles and abstracts are assessed for eligibility based on predefined criteria followed by a detailed evaluation of the full texts of potentially eligible studies. Eligible studies are peer-reviewed articles, clinical studies, or books published in English between January 1, 1992, and January 1, 2024, involving human subjects diagnosed with NPD, and examining psycholinguistic aspects of NPD. Data extraction of key information from each selected study is performed, such as sample characteristics, study design, and main findings. Finally, the results are synthesized, and a qualitative analysis identifies common psycholinguistic patterns and communicative behaviors associated with NPD.
3. Gathering neuroscientific correlates: The second systematized review extends to neuroscientific studies exploring the neural correlates of narcissistic behavior. PubMed, PubMed Central, and PsycINFO databases are searched using keywords such as “Narcissistic Personality Disorder,” “brain structures,” “functional connectivity,” and “neurobiological factors” to identify relevant studies. It scrutinizes neuroscientific findings associated with NPD.

By combining these approaches, the study aims to provide a deeper understanding of the psycholinguistic and neurobiological aspects of NPD, emphasizing the role of language in elucidating its cognitive and emotional dimensions.

## 3 Results

At the heart of NPD lie nine distinctive criteria, outlined in the *Diagnostic and Statistical Manual of Mental Disorders: Fifth Edition Text Revision DSM-5-TR*™ (American Psychiatric Association, 2022). These criteria encapsulate the ways in which narcissism permeates an individual's thoughts, feelings, and actions. The following list dissects each criterion, unveiling the behavioral and psychological facets of NPD, along with potential psycholinguistic manifestations that can be explored:

1. Grandiose sense of self-importance: Individuals with NPD often overestimate their capabilities, set unreasonably high standards for themselves, and engage in bragging or exaggerating their achievements. Linguistic markers of grandiosity might include frequent self-promotion, use of boastful language, and a tendency to downplay the accomplishments of others.
2. Frequent fantasies of success and power: Individuals with NPD harbor fantasies of possessing success, power, intelligence, beauty, love, and self-fulfillment. This might be evident in the use of language that emphasizes dominance, control, and superiority.
3. Belief in superiority: Those with NPD consider themselves special or unique, associating only with individuals they deem worthy. Psycholinguistic analysis might identify a sense of entitlement reflected in their language choices, such as making constant demands or expecting preferential treatment.
4. Need for admiration: Fragile self-esteem leads to frequent self-doubt and preoccupation with others' opinions, driving a constant need for admiration and compliments. This might manifest in seeking excessive compliments or validation through language, potentially employing manipulative tactics or subtle flattery to gain admiration.
5. Entitlement: Individuals with NPD possess an inflated sense of self-worth, expecting favorable treatment to an unreasonable degree, and expressing anger when their expectations are not met. Linguistic markers of entitlement could include a sense of superiority reflected in word choice and a tendency to make unreasonable demands.
6. Willingness to exploit others: Conscious or unconscious exploitation characterizes NPD, as individuals form relationships with those boosting their self-esteem or status, deliberately taking advantage of others. This could be reflected in persuasive language used to manipulate others or a lack of empathy for the consequences of their actions.
7. Lack of empathy: NPD entails saying things that may hurt others, viewing others' feelings as a sign of weakness, and withholding kindness or interest. Language analysis might reveal a focus on self-interest, an inability to acknowledge the emotions of others, and a dismissive attitude toward others' feelings.
8. Frequent envy: A persistent feeling of envy toward others' success, coupled with an expectation of receiving envy, defines this criterion. Psycholinguistic analysis could explore how they express envy or how they perceive others' reactions to them.
9. Arrogance: Patronizing behavior, snobbishness, and condescension are typical manifestations of arrogance in individuals with NPD. This might be reflected in condescending language, a lack of respect for others, and a tendency to belittle or dismiss others' opinions.

Accordingly, by integrating established criteria and psycholinguistic analysis, a critical examination of how language both reflects and shapes the cognitive and emotional dimensions of narcissistic behavior can be provided.

### 3.1 Psycholinguistic analysis

**Figure 1:**
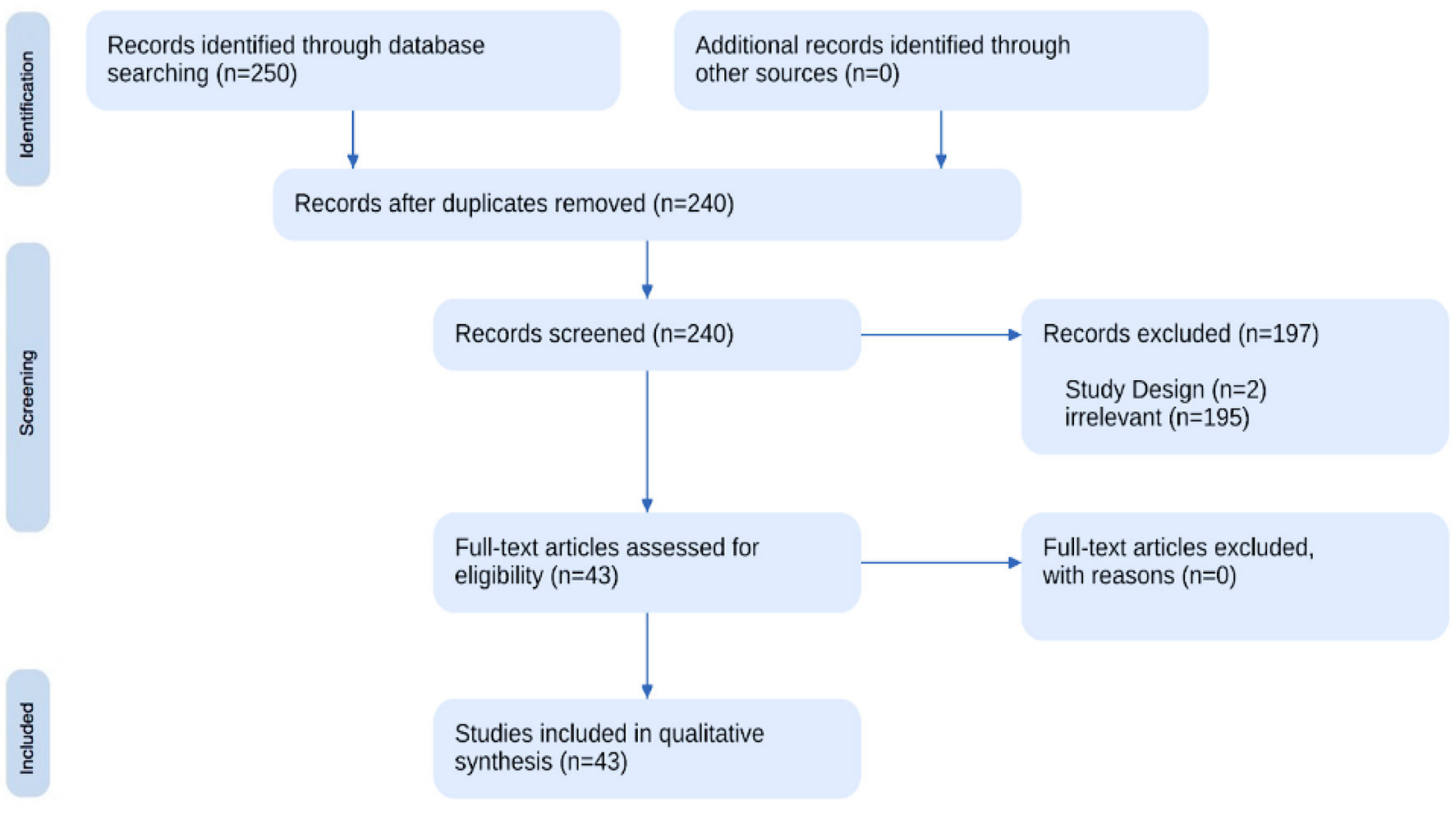
Flowchart of the psyhcolinguistic analysis review.

The first systematized review exploring psycholinguistic patterns and communicative behaviors associated with NPD identified a total of 43 relevant studies. As portrayed in Figure 1, the initial database search yielded 240 potential studies after removing 10 duplicates, which were then subject to a two-step screening process. During the first step, titles and abstracts were screened, resulting in the exclusion of 197 studies that did not meet the predefined criteria. The final selection comprised 43 studies that met the eligibility criteria (see Table A1).

The synthesis of the reviewed studies unveils psycholinguistic patterns that reflect the cognitive and emotional characteristics of NPD. Notably, identifying NPD types and distinguishing between grandiose and vulnerable narcissism has been a central focus for scholars, with clear differences emerging in communication behaviors (Miller et al., 2013, 2016; Krusemark et al., 2015, 2018; Crowe et al., 2016; Stanton and Zimmerman, 2018; Gabbard, 2022). For instance, Coleman et al. (2022) found that grandiose narcissists exhibit impulsivity in decision-making, often leading to abrupt or aggressive communicative behaviors. This impulsivity contrasts with De Panfilis et al. (2019), who observed that individuals with higher NPD traits quickly recognize neutral and low-intensity negative emotions, affecting their social interactions by selectively reinforcing their grandiose self-perception. The interrelatedness between narcissistic traits and interpersonal dysfunction is further highlighted by Day et al. (2021), whose study revealed that narcissistic traits lead to communication patterns characterized by abuse, mutual idealization, and devaluation in intimate relationships, and aligns with Nook et al.'s (2022) cognitive-behavioral model, suggesting that such traits serve as regulatory mechanisms for self-esteem. This is consistent with Grove et al.'s (2019) findings using the Narcissistic Admiration and Rivalry Questionnaire, which reveals how admiration and rivalry manifest in narcissistic communication, impacting social interactions, and relational stability.

Additionally, the psycholinguistic analysis covers the relationship between grandiose narcissism and self-esteem. For instance, Hyatt et al. (2018) identified a moderate positive correlation between them, noting that callousness, entitlement, and demeaning attitudes often surface in communication to maintain superiority and control. Caligor and Stern (2020) further explored this, suggesting that grandiose narcissists' stable self-functioning yet lacking normal identity formation results in self-aggrandizing communication patterns, while Schalkwijk et al. (2021) discussed the self-impairments in identity and self-direction, as well as interpersonal dysfunctioning, as per recent frameworks. Additionally, Kałużna-Wielobób et al. (2020) observed negative correlations between narcissism and community feeling, highlighting isolationist and anti-community sentiments in narcissistic discourse. In contrast, vulnerable narcissism tends to produce more emotionally charged and reactive language, reflecting underlying instability (Gore and Widiger, 2016; Euler et al., 2018; Rogier and Velotti, 2018; Borráz-León et al., 2023). Ponzoni et al. (2021) linked this subtype to emotion dysregulation, while Kacel et al. (2017) noted psychological distress manifesting in disorganized or defensive communication.

Moreover, empathy deficits have significant implications for communication. Studies indicate that individuals with NPD, particularly those with affective empathy deficits (Ritter et al., 2011; Morey and Stagner, 2012; Marcoux et al., 2014; Jacobs, 2022), display language that reflects a lack of regard for others' feelings (Dowson, 1992). Ronningstam (2014) emphasized that these deficits hinder self-disclosure and compromise therapeutic progress. Furthermore, the complex nature of self-esteem in NPD is evident in Mitra (2023) and Vater et al. (2013) who found lower explicit but higher implicit self-esteem, suggesting a disconnect between conscious self-perception and unconscious self-worth. This dichotomy aligns with Ronningstam's (2014) observations of challenges in self-disclosure. Arble and Barnett (2017) explored self-object needs influencing communication styles, while Janusz et al. (2021) examined control dynamics in couple therapy with NPD, revealing specific communicative strategies. Tanzilli and Gualco (2020) further underscored how different NPD subtypes elicit distinct emotional reactions from therapists, affecting therapeutic communication and alliance. Hence, communication difficulties in NPD extend beyond empathy deficits to challenges in understanding others' mental states. This finding is affirmed by Bilotta et al. (2018) who found poorer mindreading abilities in individuals with NPD, suggesting that their communication issues may be more deeply rooted in cognitive deficits.

The review also revealed various tools and approaches that have been developed and tested to diagnose and treat NPD. For instance, the Five-Factor Narcissism Inventory (FFNI) and Narcissistic Personality Inventory (NPI) have been validated as a reliable tool for assessing grandiose narcissism (Miller et al., 2014; Braun et al., 2016). However, NPI limitations, particularly regarding female narcissism, have been noted (Green et al., 2022), which further confirms sex differences in NPD symptom expression highlighted by Hoertel et al. (2018). Hence, language use may vary between genders, which also warrants further investigation and refinement in diagnostic criteria. As for therapeutic interventions, transfer-focused therapy (Weinberg and Ronningstam, 2022) and self-compassion strategies (Kramer et al., 2017), have shown promise in addressing the distorted language patterns of individuals with NPD. These interventions aim not only to mitigate distorted communication but also to help build more constructive interpersonal interactions.

This review underscores the psycholinguistic aspects of NPD, connecting language use with empathy, self-esteem, and narcissism subtypes. However, a significant gap remains in understanding the neural mechanisms underlying these psycholinguistic patterns. While studies like Preston et al. (2020) hint at potential neurological factors, further research is needed to unveil these connections. Therefore, a separate review that focuses on the neuroscientific correlates of NPD is essential to bridge this gap.

### 3.2 Neuroscientific correlates

**Figure 2:**
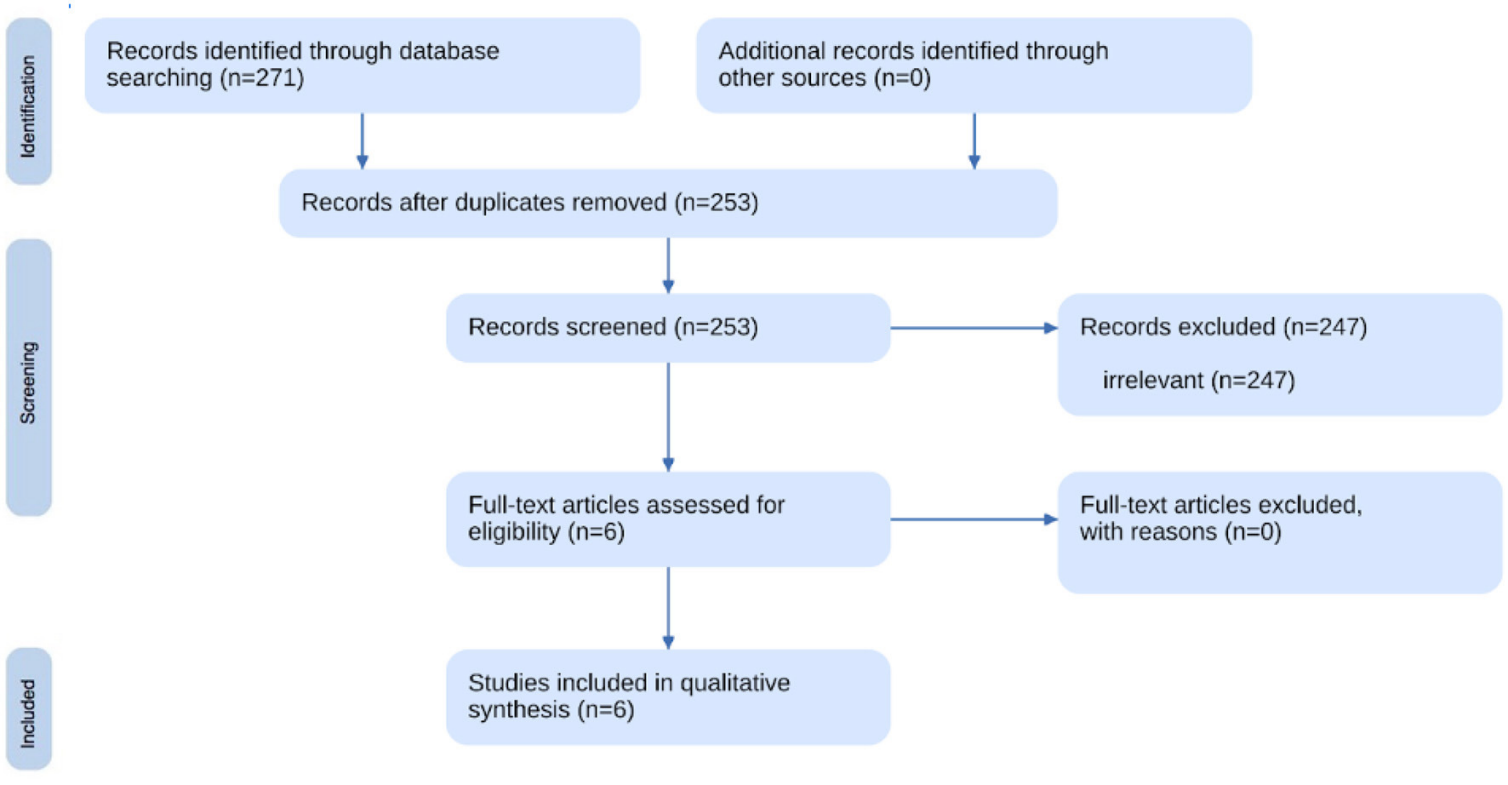
Flowchart of the neuroscientific correlates review.

The second systematized review identified a total of six relevant studies exploring examining brain structures and functions associated with NPD. The initial database search yielded 253 potential studies after removing duplicates, which were then subjected to a two-step screening process (see Figure 2). Titles and abstracts were screened, resulting in the exclusion of 247 studies that did not meet predefined criteria. The final selection comprised six studies that met the eligibility criteria (see Table A2).

The systematized review delves into the neural correlates of narcissistic personality traits using various neuroscientific methodologies. For instance, Fan et al. (2011) explored the neural mechanisms underlying emotional empathy in individuals with high narcissistic traits. They found that these individuals exhibited higher degrees of alexithymia and decreased deactivation in the right anterior insula during empathy tasks, suggesting altered neural responses to emotional stimuli in individuals with NPD. This reduced activation was suggested to be linked to greater alexithymia in pathological narcissism, indicating diminished emotional awareness and processing in individuals with narcissistic traits. Additionally, Ash et al. (2023) investigated the neural correlates of self-awareness and recognition in individuals with narcissistic traits. They identified deficits in cortical thickness, gray matter volume, and frontostriatal connectivity using various neuroimaging techniques. These structural deficits were associated with impaired social cognition and emotion regulation, suggesting a neurobiological basis for the behavioral and linguistic manifestations observed in NPD.

Subsequently, Schulze et al. (2013) investigated gray matter volume differences in individuals with NPD compared to healthy controls. They identified reductions in gray matter volume in the anterior insula, rostral anterior cingulate cortex, and dorsolateral prefrontal cortex (PFC), which inversely correlated with self-reported emotional empathy. Whole-brain analyses highlighted smaller gray matter volumes in fronto-paralimbic regions, implicating deficits in emotional and cognitive regulation. Similarly, Nenadić et al.'s (2021) study explored the relationship between subclinical narcissism and regional brain volumes using high-resolution structural magnetic resonance imaging. They found significant positive correlations between NPI scores and gray matter in multiple prefrontal cortical areas, including the medial and ventromedial prefrontal cortex, anterior/rostral dorsolateral prefrontal cortex, orbitofrontal cortex, and insula. These findings not only reinforce the involvement of prefrontal and insular regions in narcissistic traits but also align with previous functional studies linking narcissism-related phenotypes such as self-enhancement and social dominance to these brain structures.

Moreover, Stolz et al. (2021) explored prosocial decision-making in individuals with NPD using functional MRI (fMRI). They found that individuals with NPD exhibited reduced consideration of prosocial motives and diminished neural activity associated with conflict monitoring compared to healthy controls. This suggests altered neural mechanisms underlying social decision-making in individuals with NPD, which may contribute to difficulties in interpersonal relationships and social interactions. Lastly, Jornkokgoud et al. (2023) employed machine learning methods to predict narcissistic traits based on brain structural organization and personality features. They found that brain structural markers, along with normal and abnormal personality features, could accurately predict narcissistic traits. Their findings suggest that specific patterns of brain structure may predispose individuals to narcissistic traits, offering a new perspective on the neurobiological basis of NPD.

Collectively, the findings from these studies highlight consistent neural correlates associated with narcissistic traits, including alterations in the anterior insula, prefrontal cortex, and frontostriatal circuits. These structural and functional abnormalities contribute to deficits in emotional empathy, self-awareness, and social cognition observed in individuals with NPD. The studies also reveal varying patterns of neural activation and structural deficits across different aspects of narcissism. This suggests that different aspects of narcissistic traits may be mediated by distinct neural mechanisms, which warrants further investigation into the heterogeneous nature of NPD.

## 4 Discussion

The synthesis of psycholinguistic analysis and neuroscientific correlates adopted in the present study underscores the interdisciplinary nature of NPD. By critically reviewing the DSM-5-TR™ characteristics and synthesizing findings from relevant studies, this research highlights the connectedness between language use, cognitive processes, and neural mechanisms in individuals with NPD. The psycholinguistic analysis revealed distinct patterns of language use associated with core features of NPD. For instance, the differentiation between grandiose and vulnerable narcissism is particularly informative for clinicians given that grandiose narcissists' impulsive and self-promoting communication contrasts with the emotionally reactive and defensive language of vulnerable narcissists. Recognizing these patterns allows for more precise identification and therapy that address the unique cognitive and emotional needs of each subtype. Additionally, there is evidence of a discrepancy between explicit and implicit self-esteem, suggesting the potential for language analysis to uncover underlying self-perception issues. This dichotomy highlights the need for psycholinguistic tools that can better capture these differences, facilitating more accurate diagnoses and therapeutic strategies. Hence, these findings highlight the importance of a psycholinguistic analysis in informing the behavioral manifestations of NPD.

Moreover, the review of neuroscientific studies identified specific brain regions and functional abnormalities associated with NPD, providing a neurobiological framework for understanding the emotional and social impairments characteristic of NPD. The observed correlations between structural abnormalities in brain regions, such as the anterior insula and prefrontal cortex, and the communication patterns of individuals with NPD can offer a neurobiological explanation for their cognitive and emotional impairments. Accordingly, the integration of psycholinguistic and neuroscientific findings alongside a critical analysis of the DSM-5-TR™ criteria has the potential to provide a robust framework for understanding the underpinnings of NPD. This interdisciplinary approach extends beyond mere description, validates the psycholinguistic findings, and highlights the importance of developing diagnostic tools and language-based assessments that consider both behavioral and neural manifestations of NPD in everyday communication. This approach can complement existing diagnostic methods based on observable behaviors, and address the specific cognitive distortions and emotional dysregulation underlying these behaviors, potentially leading to a more accurate and timely treatment.

In addition, this interdisciplinary approach can significantly improve identification, especially for individuals who may not exhibit overt behavioral manifestations. For instance, interventions that address the underlying cognitive distortions reflected in language use can be customized to meet the specific needs of individuals with NPD by incorporating techniques that promote self-compassion and emotional regulation. Informed by the identified neural deficits, this approach can potentially enhance communication, empathy, and social interactions and help in the development of neurotherapeutic interventions that stimulate brain activity in regions associated with empathy and social cognition such as the prefrontal cortex and anterior insula. Consequently, enhancing emotional regulation and social cognition abilities in affected individuals promotes self-compassion, a crucial step in addressing the underlying emotional vulnerability often masked by the grandiosity criterion in NPD. Therefore, in order to increase the effectiveness of diagnostic tools and therapies, clinicians and researchers are encouraged to include psycholinguistic and neuroscientific perspectives in developing interdisciplinary protocols and treatments that take into account the varying profiles of NPD patients.

### 4.1 Limitations

This study has limitations that warrant consideration in future research. The reliance on existing literature and case studies may entail potential biases. Additionally, the dynamic and heterogeneous nature of NPD necessitates ongoing research to capture its evolving manifestations and refine diagnostic criteria. Thus, future research needs to focus on longitudinal case studies and large-scale multicenter collaborations to further elucidate the psycholinguistic and neurobiological underpinnings of NPD.

## 5 Conclusion

NPD is a complex psychological condition that significantly impacts individuals' self-perception and interpersonal relationships. This study combines psycholinguistic and neuroscientific approaches to provide a multidisciplinary exploration of NPD. The diagnostic criteria proposed by the American Psychiatric Association's DSM-5-TR™ were first identified and pertinent psycholinguistic and neuroscientific studies were reviewed using PubMed, PubMed Central, and PsycINFO databases. The psycholinguistic analysis identifies distinctive patterns of self-promotion and manipulative behaviors, reflecting the disorder's core features. Concurrently, neuroscientific findings reveal structural and functional abnormalities in the anterior insula and prefrontal cortex, linked to deficits in empathy, self-awareness, and social cognition. By synthesizing these findings, this study highlights the need for integrating psycholinguistic and neuroscientific insights to address the complexities of this disorder. Clinically, this interdisciplinary approach can guide the development of patient-centered therapeutic approaches, potentially improving communication, empathy, and social interactions in individuals with NPD.

## Author contributions

DE: Conceptualization, Methodology, Writing – original draft, Writing – review & editing.
