# Supplementary material for: Narcissistic Personality Disorder through psycholinguistic analysis and neuroscientific correlates
Source: Front Behav Neurosci. 2024 Jul 17;18:1354258. doi: 10.3389/fnbeh.2024.1354258 (PMC11299496; doi:10.3389/fnbeh.2024.1354258)
Supplement: Supplementary file 1 [file Table_1.docx]

APPENDIX A

Table A1

| **First Author** | **Year** | **Title** | **Participants** | **Intervention/Study Design** | **Findings** |
| --- | --- | --- | --- | --- | --- |
| Dowson, J H | 1992 | DSM-III-R narcissistic personality disorder evaluated by patients’ and informants’ self-report questionnaires: relationships with other personality disorders and a sense of entitlement as an indicator of narcissism. | Patients and their informants (60 patients in total) | Modified versions of the revised Personality Diagnostic Questionnaire (PDQ-R) for DSM-III-R personality disorders | Relationship between NPD scores and other PDs, and reliability indicators. Patient self-evaluation of sense of entitlement showed satisfactory reliability, indicating its potential as an indicator of narcissism. |
| Ritter, Kathrin | 2011 | Lack of empathy in patients with narcissistic personality disorder. | Patients with NPD, healthy controls, clinical controls with BPD | Assessment of emotional and cognitive empathy with MET and MASC | Significant impairments in emotional empathy on MET, no deficits in cognitive empathy on MET or MASC. |
| Morey, Leslie C | 2012 | Narcissistic pathology as core personality dysfunction: comparing the DSM-IV and the DSM-5 proposal for narcissistic personality disorder. | Patients with NPD | Comparison of DSM-IV and proposed DSM-5 criteria for personality disorders | The study examines the proposed DSM-5 criteria for NPD, emphasizing shared deficits among personality disorders, such as empathic capacity deficits. |
| Vater, Aline | 2013 | When grandiosity and vulnerability collide: Implicit and explicit self-esteem in patients with narcissistic personality disorder. | Patients with NPD | Implicit and explicit self-esteem assessments | Patients with NPD scored lower on explicit self-esteem compared to non-clinical controls. They scored higher on both explicit and implicit self-esteem compared to patients with BPD. Damaged self-esteem (low explicit, high implicit) was associated with higher narcissistic psychopathology within the NPD group. |
| Miller, Joshua D | 2013 | Grandiose and vulnerable narcissism and the DSM-5 pathological personality trait model. | Patients with NPD | Examination of relations among traits from the Personality Inventory for DSM-5 (PID5) and grandiose and vulnerable narcissism | The 25 traits from PID5 captured a significant portion of the variance in grandiose and vulnerable factors, with specific facets designated for NPD assessment performing better in assessing grandiose rather than vulnerable narcissism. |
| Marcoux, Louis-Alexandre | 2014 | Feeling but not caring: empathic alteration in narcissistic men with high psychopathic traits. | Patients with severe narcissistic personality disorder (NPD, n=11) and community controls (CC, n=13) | Comparison of somatosensory responses to pain and observation of pain between NPD and CC subjects | Differences in pain thresholds and somatosensory gating (SG) during anticipation and observation of pain in others. |
| Ronningstam, Elsa | 2014 | Beyond the diagnostic traits: a collaborative exploratory diagnostic process for dimensions and underpinnings of narcissistic personality disorder. | Patients with severe narcissistic personality disorder (NPD, n=11) and community controls (CC, n=13) | - | Diagnosing NPD is challenging in psychiatric and clinical practice due to patients’ limited ability to recognize their impact on others, hypersensitivity, defensive reactivity, and compromised self-disclosure and empathy. |
| Miller, Joshua D | 2014 | A comparison of the criterion validity of popular measures of narcissism and narcissistic personality disorder via the use of expert ratings. | 98 community participants receiving psychological/psychiatric treatment | Comparison of multiple narcissism measures to expert consensus ratings of personality traits associated with NPD according to DSM-IV-TR and 5-factor model traits | Five-Factor Narcissism Inventory (FFNI) and Narcissistic Personality Inventory (NPI) grandiose dimensions matched strongest with expert ratings of DSM-IV-TR NPD and grandiose narcissism. |
| Krusemark, Elizabeth A | 2015 | Narcissism dimensions differentially moderate selective attention to evaluative stimuli in incarcerated offenders. | Male offenders with NPD traits | Differentiating between grandiose and vulnerable narcissism | Vulnerable narcissism associated with attention biases for positive and negative stimuli, disengagement difficulties, greater recognition memory bias for negative words; Grandiose narcissism associated with increased accuracy for positive stimuli and attention away from negative stimuli. |
| Braun, Stéphanie | 2016 | French Adaptation of the Narcissistic Personality Inventory in a Belgian French-Speaking Sample. | Belgian French-speaking students (n = 942) | Administration of the Narcissistic Personality Inventory (NPI) | The total score of the NPI was validated as a self-report measure for Narcissistic Personality Disorder in its grandiose form. Future |
| Crowe, Michael | 2016 | Validation of the Narcissistic Grandiosity Scale and creation of reduced item variants. | Three samples comprising participants | Various measures and models were used for validation (e.g., grandiose and vulnerable narcissism scales, FFM, interpersonal circumplex, self-esteem measures, PID-5) | The Narcissistic Grandiosity Scale was found to be a unidimensional measure with good convergent, discriminant, and criterion validity. |
| Gore, Whitney L | 2016 | Fluctuation between grandiose and vulnerable narcissism. | Three samples comprising undergraduate students and community members oversampled for narcissistic features. | Fluctuations in grandiose and vulnerable states | Moderate association between dispositional and state narcissism. Dispositionally grandiose individuals show both grandiosity and vulnerability. Dispositionally vulnerable individuals show high vulnerability and low grandiosity. |
| Miller, Joshua D | 2016 | Rejoinder: A Construct Validity Approach to the Assessment of Narcissism. | Individuals with characteristics of grandiose narcissism | Conceptualization of grandiose narcissism tied to clinical observations | PNI-G requires revision; should be buttressed by other scales when being used as a measure of grandiose narcissism |
| Kacel, Elizabeth L | 2017 | Narcissistic Personality Disorder in Clinical Health Psychology Practice: Case Studies of Comorbid Psychological Distress and Life-Limiting Illness. | Individuals with NPD | Observation and assessment of core features | Persistent pattern of grandiosity, fantasies of unlimited power or importance, need for admiration or special treatment, psychological distress, functional impairment, poor prognosis in therapy, slow progress to behavioral change, premature termination, negative therapeutic alliance |
| Arble, Eamonn | 2017 | An Analysis of Self: The Development and Assessment of a Measure of Selfobject Needs. | 686 and 672 respondents in two separate studies | Development of the Arble Estimate of Selfobject Pursuits (AESOP) | Self-report measure assessing idealizing, mirroring, and twinship selfobject needs identified by Kohut; exploratory and confirmatory factor analyses, model fit, and validity analyses |
| Hyatt, Courtland S | 2018 | Narcissism and self-esteem: A nomological network analysis. | Individuals with narcissism and self-esteem (Combined sample of 4711) | Developmental experiences, individual differences, interpersonal functioning, psychopathology | Small to moderate positive correlation (r = .28) between grandiose narcissism and self-esteem; grandiose narcissism related to callousness, grandiosity, entitlement, and demeaning attitudes towards others, negatively associated with internalizing psychopathology and generally unrelated to externalizing behaviors |
| Bilotta, Elena | 2018 | Symptom severity and mindreading in narcissistic personality disorder. | Narcissistic patients (NPD); Patients with PD; Patients without PD | Comparison of mindreading capacities and symptomatic distress | NPD patients showed poorer mindreading capacities compared to patients without PD, similar to patients with other PDs. Symptomatic subjective distress in NPD was less severe than in other PDs and comparable to patients without PD. |
| Krusemark, Elizabeth A | 2018 | Comparing self-report measures of grandiose narcissism, vulnerable narcissism, and narcissistic personality disorder in a male offender sample. | Male offenders with NPD traits | The study utilized self-report measures of grandiose narcissism (Narcissistic Personality Inventory-13 [NPI-13]), vulnerable narcissism (Hypersensitive Narcissism Scale [HSNS]), and narcissistic personality disorder (NPD; Personality Diagnostic Questionnaire [PDQ]) | The Narcissistic Personality Inventory-13 (NPI-13) and Personality Diagnostic Questionnaire (PDQ) both showed substantial overlap (rICC = .81) and significant correlations with interview-based symptoms of NPD, entitlement, psychopathy, and externalizing behaviors, while the NPI-13 demonstrated incremental validity in uniquely explaining variance related to NPD criteria, indicating its efficacy in assessing grandiose narcissism. |
| Euler, Sebastian | 2018 | Grandiose and Vulnerable Narcissism in Borderline Personality Disorder. | Borderline Personality Disorder (BPD) patients (n = 65) | Completion of the Pathological Narcissism Inventory and psychometric measures for impulsivity, anger, borderline symptom severity, personality organization, depression, and rejection sensitivity | Grandiose narcissism showed a stronger association with NPD than with BPD (p < 0.01) - Vulnerable narcissism was only associated with BPD (p < 0.01) |
| Rogier, Guyonne | 2018 | Narcissistic Implications in Gambling Disorder: The Mediating Role of Emotion Dysregulation. | Addicted gamblers (n = 74), Healthy controls (n = 105) | South Oaks Gambling Screen (SOGS), Pathological Narcissism Inventory (PNI), Difficulties in Emotion Regulation Scale (DERS) | Differences in PNI and DERS scores between addicted gamblers and healthy controls. Higher levels of vulnerable narcissism among strategic addicted gamblers. Emotion dysregulation mediates the relationship between grandiose narcissism and GD severity. |
| Hoertel, Nicolas | 2018 | Examining sex differences in DSM-IV-TR narcissistic personality disorder symptom expression using Item Response Theory (IRT). | Large, nationally representative sample from the USA (n=34,653) | Item response theory methods to examine sex differences in DSM-IV-TR NPD symptoms | Statistically and clinically significant sex differences for 2 out of 9 DSM-IV-TR NPD symptoms: 1. Males more likely to endorse ’lack of empathy’ at lower levels of NPD severity than females. 2. ’Being envious’ better indicator of NPD severity in males than females. No clinically significant sex differences on the remaining NPD symptoms. |
| Kramer, Ueli | 2018 | The role of shame and self-compassion in psychotherapy for narcissistic personality disorder: An exploratory study. | Patients with NPD | Long-term clarification-oriented psychotherapy | Decrease in frequency of shame from Session 25 to Session 36 (d = .30); presence of self-compassion in Session 36 linked with specific therapist interventions (process-guidance and treatment of behavior-underlying assumptions, explaining 51% of variance) |
| Stanton, Kasey | 2018 | Clinician ratings of vulnerable and grandiose narcissistic features: Implications for an expanded narcissistic personality disorder diagnosis. | Adult outpatients with NPD (N = 2,149) | Examination of clinician ratings of traits related to grandiosity and vulnerability within NPD | Structural results indicated that personality features related to vulnerability (e.g., perfectionism, inadequacy) were unrelated to ratings of grandiose narcissistic features. Scores on configurations defined by grandiose narcissistic traits related positively to psychosocial impairment indicators; configurations with vulnerable features showed stronger relations with impairment. |
| Grove, Jeremy L | 2019 | Narcissistic Admiration and Rivalry: An Interpersonal Approach to Construct Validation. | Undergraduate students | Self-report measures of interpersonal processes based in the Interpersonal Circumplex (IPC) | Examination of the construct validity of the Narcissistic Admiration and Rivalry Questionnaire (NARQ) based on the interpersonal perspective |
| De Panfilis, Chiara | 2019 | Facial Emotion Recognition and Social-Cognitive Correlates of Narcissistic Features. | Non-clinical individuals with self-reported NPD features | Facial Emotion Recognition (FER) task assessing emotional intensity | Individuals with higher NPD features were faster at recognizing neutral and 25%-intensity negative emotions. This response pattern mediated the association between NPD features and increased anger about rejection. |
| Kałużna-Wielobób, Alina | 2020 | Community Feeling and Narcissism as Two Opposite Phenomena. | University students (N = 520) | Completion of CFQ, NARQ, and HSNS questionnaires | Structural equation modeling confirmed negative relations between narcissism (both grandiose and vulnerable) and community feeling, and positive relations with anti-community domination and isolation. Community feeling and narcissism are related constructs but not reducible to each other. |
| Preston, Stephanie D | 2020 | Understanding empathy and its disorders through a focus on the neural mechanism. | Individuals with Borderline Personality Disorder (BPD), Narcissistic Personality Disorder (NPD), and Frontotemporal Dementia (FTD) | Examination of emotional and cognitive empathy components and their overlap | Impaired empathy at emotional and cognitive stages, underlying causes of empathy disorders, misinterpretation of survey and task results, clarification of mechanisms and interdependence of emotional and cognitive empathy, integration of experimental results |
| Caligor, Eve | 2020 | Diagnosis, Classification, and Assessment of Narcissistic Personality Disorder Within the Framework of Object Relations Theory. | Individuals diagnosed with NPD | Comparison between the Object Relations Theory (ORT) model and the Alternative Model for Personality Disorders (AMPD) in DSM-5 diagnostic criteria | ORT defines NPD with specific self-pathology, reflecting a grandiose self-structure within borderline personality organization. The model highlights stability of self-functioning but lack of normal identity formation and reliance on maintaining a sense of exceptionalism. Significant correspondence with AMPD criteria noted, alongside conceptual differences. |
| Tanzilli, Annalisa | 2020 | Clinician Emotional Responses and Therapeutic Alliance When Treating Adolescent Patients With Narcissistic Personality Disorder Subtypes: A Clinically Meaningful Empirical Investigation. | Adolescent patients with specific subtypes of Narcissistic Personality Disorder (NPD) | Therapist Response Questionnaire for Adolescents, Working Alliance Inventory, Shedler-Westen Assessment Procedure-II for Adolescents | Grandiose subtype: positively related to angry/criticized and disengaged/hopeless responses; negatively related to warm/attuned response. Fragile subtype: positively related to overinvolved/worried response. High-functioning/exhibitionistic subtype: negatively related to angry/criticized response. Lower quality of therapeutic alliance positively associated with grandiose subtype. Empirically founded prototypes of therapist responses to NPD subtypes resemble theoretical-clinical accounts. |
| Rosenthal, Seth A | 2020 | The Narcissistic Grandiosity Scale: A Measure to Distinguish Narcissistic Grandiosity From High Self-Esteem. | Individuals with narcissistic traits | Evaluation of shortened versions of the Narcissistic Grandiosity Scale (NGS) | Correlations of NGS items with grandiose narcissism, distinguishing from self-esteem and narcissistic entitlement |
| Schalkwijk, Frans | 2021 | Narcissistic Personality Disorder: Are Psychodynamic Theories and the Alternative DSM-5 Model for Personality Disorders Finally Going to Meet? | Individuals with NPD | Examination of recent developments and conceptualizations of NPD, including relational and intersubjective psychoanalytic perspectives, the Alternative DSM-5 Model for Personality Disorders (AMPD), and the hierarchical model of pathological narcissism by Pincus and Lukowitsky | Evaluation of the implications of these frameworks on understanding the self-impairments in identity and self-direction, as well as interpersonal dysfunctioning in individuals with NPD |
| Janusz, Bernadetta | 2021 | Practices of Claiming Control and Independence in Couple Therapy With Narcissism. | Four couple therapy first consultations | Sociologically enriched and broadened concept of narcissistic disorder based on Goffman’s micro-sociology of the self | Controlling practices observed during therapy sessions, including resistance to answering therapist’s questions, blocking or dominating conversation topics, and displaying interactional independence |
| Day, Nicholas J S | 2021 | Pathological narcissism: An analysis of interpersonal dysfunction within intimate relationships. | Individuals in relationships with relatives high in narcissistic traits (N = 436) | Thematic analysis of participant responses | Abuse from relative with narcissism (physical, verbal, emotional, sexual), challenging financial and sexual behaviors, mutual idealization and devaluation, anxiety, depression, self-aggression, sickness, somatic concerns, overt hostility, dependency strivings, frustrated dependency themes |
| Ponzoni, Sara | 2021 | Emotion dysregulation acts in the relationship between vulnerable narcissism and suicidal ideation. | Individuals with suicide ideation (n = 70) and community participants (n = 154) | Administered PNI, DERS, PID-5-BF, and BSI | Significant correlation between BSI scores and vulnerable narcissism; no correlation with grandiose narcissism; correlation between BSI scores and all DERS dimensions except Awareness; emotion dysregulation moderates the relationship between vulnerable narcissism and suicide ideation; relationship between vulnerable narcissism and suicide ideation is mediated by emotion dysregulation |
| Gabbard, Glen O | 2022 | Narcissism and suicide risk. | Individuals with extreme and pathological narcissism | Classification into grandiose/oblivious, vulnerable/hypervigilant, and high-functioning subtypes, study of biological and psychological factors, and evaluation of suicide risk | Identification of three subtypes of NPD: grandiose/oblivious, vulnerable/hypervigilant, and high-functioning subtypes. Identification of both biological and psychological factors contributing to pathological narcissism, and elevated suicide risk in individuals with NPD, particularly following severe narcissistic injury |
| Jacobs, Kerrin A | 2022 | The concept of Narcissistic Personality Disorder-Three levels of analysis for interdisciplinary integration. | Individuals with NPD | Description and understanding of deficient empathy as: 1) disturbed interpersonal functioning and lack of recognition, 2) psychic disintegration and dissocial aspects, and 3) dysfunctional affective empathy and mind-reading abilities | Deficient empathy in NPD is characterized by: 1) disturbed interpersonal functioning, understood as a lack of recognition of others’ emotions and experiences, 2) psychic disintegration, particularly in dissocial aspects, affecting their ability to empathize with others on a deeper emotional level, and 3) dysfunctional affective empathy and impaired mind-reading abilities in individuals with NPD correlate with somatic changes and contribute to overall empathy impairments. |
| Nook, Erik C | 2022 | A Cognitive-Behavioral Formulation of Narcissistic Self-Esteem Dysregulation. | Individuals diagnosed with NPD | Introduction of a cognitive-behavioral model of narcissistic self-esteem dysregulation | Symptoms of NPD conceptualized as cognitive and behavioral habits regulating emotions from maladaptive self-esteem beliefs and interpretations |
| Coleman, Sulamunn R M | 2022 | Delay discounting and narcissism: A meta-analysis with implications for narcissistic personality disorder. | Adults with psychiatric conditions | Meta-analysis examining associations between DD and narcissism | Positive association between DD and narcissism (r = .21; 95% CI [.10, .32]), specifically trait grandiosity (r = .24; 95% CI [.11, .37]) |
| Green, Ava | 2022 | Female Narcissism: Assessment, Aetiology, and Behavioural Manifestations. | Individuals with narcissistic traits | Diagnostic assessment and conceptualization of NPD, review of aetiological factors, and examination of aggression and partner violence perpetration | The review critiques the DSM-5 and NPI for their limited applicability to females, emphasizes the need to understand narcissistic manifestations in women through a lens that aligns with male-defined narcissism, and identifies gender-specific patterns in aggression and partner violence perpetration. |
| Weinberg, Igor | 2022 | Narcissistic Personality Disorder: Progress in Understanding and Treatment. | Individuals with NPD | Assessment of treatment approaches compared to no treatment, revealing common treatment elements such as clear goals, attention to treatment frame, relationship and self-esteem focus, alliance building, and countertransference monitoring. | The identification of mechanisms (self-esteem dysregulation, emotion dysregulation, cognitive style, interpersonal relations, empathy) among individuals with NPD, compared to normal psychological functioning, supports a multifactorial etiology and pathogenesis of NPD, with mechanisms associated with each area of dysfunction. |
| Borráz-León, Javier I | 2023 | Cortisol reactivity to psychosocial stress in vulnerable and grandiose narcissists: An exploratory study. | Subjects with high narcissism scores (both grandiose and vulnerable) | Trier Social Stress Test (TSST), Saliva samples for cortisol levels, Personality questionnaires | Higher emotional and cortisol response in vulnerable narcissism; Positive correlation between vulnerable narcissism and schizotypal traits; Positive correlation between grandiose narcissism and psychopathic traits |
| di Giacomo, Ester | 2023 | The dark side of empathy in narcissistic personality disorder. | Individuals with NPD | Narrative review of empathic attitude, systematic search of literature on NPD and empathy. | People with NPD exhibit characteristics of self-absorption, grandiosity, and exploitation of others, with impairments primarily in affective empathy while cognitive empathy remains relatively preserved, which may contribute to therapeutic improvements. |
| Mitra, Paroma | 2023 | Narcissistic Personality Disorder | Individuals with NPD | Interventions for Narcissistic Personality Disorder (NPD) primarily focus on psychotherapy, particularly transfer-focused therapy, as pharmacotherapy has shown limited efficacy. | Findings across various studies suggest that NPD is associated with specific temperament traits such as low harm avoidance, high novelty seeking, and high reward dependence, which contribute to persistent maladaptive behaviors and impaired social functioning. |

Table A2

| **First Author** | **Year** | **Title** | **Participants** | **Intervention/Study Design** | **Findings** |
| --- | --- | --- | --- | --- | --- |
| Fan, Yan | 2011 | The narcissistic self and its psychological and neural correlates: an exploratory fMRI study. | Healthy subjects with high (n=11) and low (n=11) narcissistic personality traits based on the Narcissism Inventory (NI) scores | Psychological, behavioral, and fMRI measurements of empathy | High narcissistic subjects showed higher scores on the Symptom Checklist-90 - Revised (SCL-90-R) and the 20-item Toronto Alexithymia Scale (TAS-20). They also exhibited significantly decreased deactivation during empathy, especially in the right anterior insula. |
| Schulze, Lars | 2013 | Gray matter abnormalities in patients with narcissistic personality disorder. | Patients with NPD (n = 17) and Healthy Controls (n = 17) | Structural brain images | Patients with NPD had smaller gray matter (GM) volume in the left anterior insula compared to healthy controls. GM volume in the left anterior insula was positively related to self-reported emotional empathy. Complementary whole-brain analyses revealed smaller GM volume in fronto-paralimbic brain regions comprising the rostral and median cingulate cortex as well as dorsolateral and medial parts of the prefrontal cortex. |
| Nenadić, Igor | 2021 | Narcissistic personality traits and prefrontal brain structure | Non-narcissistic individuals, as well as participants with both grandiose and vulnerable narcissism. | Brain imaging was conducted to identify altered brain regions and networks associated with narcissism. | Brain regions associated with grandiose narcissism may also be linked to an intense desire for fame, while those associated with vulnerable narcissism may resemble those found in celebrity worshipers. |
| Stolz, David S | 2021 | Reduced frontal cortical tracking of conflict between self-beneficial versus prosocial motives in Narcissistic Personality Disorder. | Patients diagnosed with NPD and healthy controls | Functional magnetic resonance imaging (fMRI) while performing prosocial decision-making task | Participants with NPD exhibited less prosocial behavior overall compared to healthy controls, demonstrated increased response variability with varying levels of motivational conflict, responded faster overall except when making prosocial choices, showed reduced neural activity in the dorsomedial prefrontal cortex during conflict monitoring, and displayed reduced consideration of prosocial motives, indicating potential for improving interpersonal relationships and supporting well-being. |
| Ash, Sydney | 2023 | The Neural Correlates of Narcissism: Is There a Connection with Desire for Fame and Celebrity Worship? | Individuals with grandiose narcissism and vulnerable narcissism. | Comparison of brain regions and networks in individuals with grandiose and vulnerable narcissism. | Increased levels of grandiose narcissism are associated with an increased desire for fame. Vulnerable narcissism is associated with celebrity worship. The frontal regions of the brain are implicated in both narcissism and these behaviors, suggesting a neurological basis. Future research is needed to confirm these relationships. |
| Jornkokgoud, Khanitin | 2023 | Predicting narcissistic personality traits from brain and psychological features: A supervised machine learning approach. | Individuals with narcissistic traits and relevant normal and abnormal personality features | Machine learning-based methods (Kernel Ridge Regression and Support Vector Regression) | Predictive model identified a circuit involving the lateral and middle frontal gyri, angular gyrus, Rolandic operculum, and Heschl’s gyrus successfully predicting narcissistic traits (p < 0.003). Narcissistic traits were also predicted by normal (openness, agreeableness, conscientiousness) and abnormal (borderline, antisocial, insecure, addicted, negativistic, machiavellianism) personality traits. This study is the first to predict narcissistic personality traits via a supervised machine learning approach. |

**References**

Arble, E., & Barnett, D. (2017, February 21). An Analysis of Self: The Development and Assessment of a Measure of Selfobject Needs. Journal of Personality Assessment, 99(6), 608–618. <https://doi.org/10.1080/00223891.2016.1278379>

Ash, S., Greenwood, D., & Keenan, J. P. (2023, October 23). The Neural Correlates of Narcissism: Is There a Connection with Desire for Fame and Celebrity Worship? Brain Sciences, 13(10), 1499. <https://doi.org/10.3390/brainsci13101499>

Bilotta, E., Carcione, A., Fera, T., Moroni, F., Nicolò, G., Pedone, R., Pellecchia, G., Semerari, A., & Colle, L. (2018, August 15). Symptom severity and mindreading in narcissistic personality disorder. PLOS ONE, 13(8), e0201216. <https://doi.org/10.1371/journal.pone.0201216>

Borráz-León, J. I., Spreitzer, A., Scrivner, C., Landers, M., Lee, R., & Maestripieri, D. (2023, January 6). Cortisol reactivity to psychosocial stress in vulnerable and grandiose narcissists: An exploratory study. Frontiers in Psychology, 13. <https://doi.org/10.3389/fpsyg.2022.1067456>

Braun, S., Kempenaers, C., Linkowski, P., & Loas, G. (2016, December 23). French Adaptation of the Narcissistic Personality Inventory in a Belgian French-Speaking Sample. Frontiers in Psychology, 7. <https://doi.org/10.3389/fpsyg.2016.01980>

Caligor, E., & Stern, B. L. (2020, March). Diagnosis, Classification, and Assessment of Narcissistic Personality Disorder Within the Framework of Object Relations Theory. Journal of Personality Disorders, 34(Supplement), 104–121. <https://doi.org/10.1521/pedi.2020.34.supp.104>

Coleman, S. R. M., Oliver, A. C., Klemperer, E. M., DeSarno, M. J., Atwood, G. S., & Higgins, S. T. (2022, May). Delay discounting and narcissism: A meta-analysis with implications for narcissistic personality disorder. *Personality Disorders: Theory, Research, and Treatment, 13(3)*, 210–220. <https://doi.org/10.1037/per0000528>

Crowe, M., Carter, N. T., Campbell, W. K., & Miller, J. D. (2016, December). Validation of the Narcissistic Grandiosity Scale and creation of reduced item variants. *Psychological Assessment, 28(12),* 1550–1560. <https://doi.org/10.1037/pas0000281>

Day, N. J. S., Townsend, M. L., & Grenyer, B. F. S. (2021, November 16). Pathological narcissism: An analysis of interpersonal dysfunction within intimate relationships. *Personality and Mental Health, 16(3),* 204–216. <https://doi.org/10.1002/pmh.1532>

De Panfilis, C., Antonucci, C., Meehan, K. B., Cain, N. M., Soliani, A., Marchesi, C., Clarkin, J. F., & Sambataro, F. (2019, August). Facial Emotion Recognition and Social-Cognitive Correlates of Narcissistic Features. *Journal of Personality Disorders, 33(4),* 433–449. <https://doi.org/10.1521/pedi_2018_32_350>

di Giacomo, E., Andreini, E., Lorusso, O., & Clerici, M. (2023, March 30). The dark side of empathy in narcissistic personality disorder. *Frontiers in Psychiatry, 14.* <https://doi.org/10.3389/fpsyt.2023.1074558>

Dowson, J. H. (1992, November). DSM-III-R narcissistic personality disorder evaluated by patients’ and informants’ self-report questionnaires: Relationships with other personality disorders and a sense of entitlement as an indicator of narcissism. *Comprehensive Psychiatry, 33(6),* 397–406. <https://doi.org/10.1016/0010-440x(92)90062-u>

Euler, S., Stöbi, D., Sowislo, J., Ritzler, F., Huber, C., Lang, U., Wrege, J., & Walter, M. (2018). Grandiose and Vulnerable Narcissism in Borderline Personality Disorder. Psychopathology, 51(2), 110–121. <https://doi.org/10.1159/000486601>

Fan, Y., Wonneberger, C., Enzi, B., de Greck, M., Ulrich, C., Tempelmann, C., Bogerts, B., Doering, S., & Northoff, G. (2011). The narcissistic self and its psychological and neural correlates: an exploratory fMRI study. *Psychological medicine, 41(8),* 1641–1650. <https://doi.org/10.1017/S003329171000228X>

Gabbard, G. O. (2022, January 22). Narcissism and suicide risk. Annals of General Psychiatry, 21(1). <https://doi.org/10.1186/s12991-022-00380-8>

Gore, W. L., & Widiger, T. A. (2016). Fluctuation between grandiose and vulnerable narcissism. Personality Disorders: Theory, Research, and Treatment, 7(4), 363–371. <https://doi.org/10.1037/per0000181>

Green, A., MacLean, R., & Charles, K. (2022). Female Narcissism: Assessment, Aetiology, and Behavioural Manifestations. Psychological Reports, 125(6), 2833–2864. <https://doi.org/10.1177/00332941211027322>

Grove, J. L., Smith, T. W., Girard, J. M., & Wright, A. G. (2019, December). Narcissistic Admiration and Rivalry: An Interpersonal Approach to Construct Validation. Journal of Personality Disorders, 33(6), 751–775. <https://doi.org/10.1521/pedi_2019_33_374>

Hoertel, N., Peyre, H., Lavaud, P., Blanco, C., Guerin-Langlois, C., René, M., Schuster, J. P., Lemogne, C., Delorme, R., & Limosin, F. (2018, February). Examining sex differences in DSM-IV-TR narcissistic personality disorder symptom expression using Item Response Theory (IRT). Psychiatry Research, 260, 500–507. <https://doi.org/10.1016/j.psychres.2017.12.031>

Hyatt, C. S., Sleep, C. E., Lamkin, J., Maples-Keller, J. L., Sedikides, C., Campbell, W. K., & Miller, J. D. (2018, August 1). Narcissism and self-esteem: A nomological network analysis. PLOS ONE, 13(8), e0201088. <https://doi.org/10.1371/journal.pone.0201088>

Jacobs, K. A. (2022, November 16). The concept of Narcissistic Personality Disorder–Three levels of analysis for interdisciplinary integration. Frontiers in Psychiatry, 13. <https://doi.org/10.3389/fpsyt.2022.989171>

Janusz, B., Bergmann, J. R., Matusiak, F., & Peräkylä, A. (2021, January 25). Practices of Claiming Control and Independence in Couple Therapy With Narcissism. Frontiers in Psychology, 11. <https://doi.org/10.3389/fpsyg.2020.596842>

Jornkokgoud, K., Baggio, T., Faysal, M., Bakiaj, R., Wongupparaj, P., Job, R., & Grecucci, A. (2023, July 31). Predicting narcissistic personality traits from brain and psychological features: A supervised machine learning approach. *Social Neuroscience, 18(5)*, 257–270. <https://doi.org/10.1080/17470919.2023.2242094>

Kacel, E. L., Ennis, N., & Pereira, D. B. (2017, July 3). Narcissistic Personality Disorder in Clinical Health Psychology Practice: Case Studies of Comorbid Psychological Distress and Life-Limiting Illness. Behavioral Medicine, 43(3), 156–164. <https://doi.org/10.1080/08964289.2017.1301875>

Kałużna-Wielobób, A., Strus, W., & Cieciuch, J. (2020, October 27). Community Feeling and Narcissism as Two Opposite Phenomena. Frontiers in Psychology, 11. <https://doi.org/10.3389/fpsyg.2020.515895>

Kramer, U., Pascual‐Leone, A., Rohde, K. B., & Sachse, R. (2017, December 19). The role of shame and self‐compassion in psychotherapy for narcissistic personality disorder: An exploratory study. Clinical Psychology & Psychotherapy, 25(2), 272–282. <https://doi.org/10.1002/cpp.2160>

Krusemark, E. A., Campbell, W. K., Crowe, M. L., & Miller, J. D. (2018, July). Comparing self-report measures of grandiose narcissism, vulnerable narcissism, and narcissistic personality disorder in a male offender sample. Psychological Assessment, 30(7), 984–990. <https://doi.org/10.1037/pas0000579>

Krusemark, E. A., Lee, C., & Newman, J. P. (2015). Narcissism dimensions differentially moderate selective attention to evaluative stimuli in incarcerated offenders. Personality Disorders: Theory, Research, and Treatment, 6(1), 12–21. <https://doi.org/10.1037/per0000087>

Marcoux, L. A., Michon, P. E., Lemelin, S., Voisin, J. A., Vachon-Presseau, E., & Jackson, P. L. (2014, December). Feeling but not caring: Empathic alteration in narcissistic men with high psychopathic traits. Psychiatry Research: Neuroimaging, 224(3), 341–348. <https://doi.org/10.1016/j.pscychresns.2014.10.002>

Miller, J. D., Gentile, B., Wilson, L., & Campbell, W. K. (2013, May). Grandiose and Vulnerable Narcissism and theDSM–5Pathological Personality Trait Model. Journal of Personality Assessment, 95(3), 284–290. <https://doi.org/10.1080/00223891.2012.685907>

Miller, J. D., Lynam, D. R., & Campbell, W. K. (2016). Rejoinder. Assessment, 23(1), 18–22. <https://doi.org/10.1177/1073191115608943>

Miller, J. D., McCain, J., Lynam, D. R., Few, L. R., Gentile, B., MacKillop, J., & Campbell, W. K. (2014, September). A comparison of the criterion validity of popular measures of narcissism and narcissistic personality disorder via the use of expert ratings. Psychological Assessment, 26(3), 958–969. <https://doi.org/10.1037/a0036613>

Mitra, P. (2023, March 13). *Narcissistic Personality Disorder*. StatPearls - NCBI Bookshelf. <https://www.ncbi.nlm.nih.gov/books/NBK556001/>

Morey, L. C., & Stagner, B. H. (2012, June 21). Narcissistic Pathology as Core Personality Dysfunction: Comparing the DSM‐IV and the DSM‐5 Proposal for Narcissistic Personality Disorder. Journal of Clinical Psychology, 68(8), 908–921. <https://doi.org/10.1002/jclp.21895>

Nenadić, I., Lorenz, C., & Gaser, C. (2021). Narcissistic personality traits and prefrontal brain structure. *Scientific Reports*, *11*(1). <https://doi.org/10.1038/s41598-021-94920-z>

Nook, E. C., Jaroszewski, A. C., Finch, E. F., & Choi-Kain, L. W. (2022, October). A Cognitive-Behavioral Formulation of Narcissistic Self-Esteem Dysregulation. *FOCUS, 20(4)*, 378–388. <https://doi.org/10.1176/appi.focus.20220055>

Ponzoni, S., Beomonte Zobel, S., Rogier, G., & Velotti, P. (2021, May 6). Emotion dysregulation acts in the relationship between vulnerable narcissism and suicidal ideation. *Scandinavian Journal of Psychology, 62(4),* 468–475. <https://doi.org/10.1111/sjop.12730>

Preston, S. D., Ermler, M., Lei, Y., & Bickel, L. (2020, June). Understanding empathy and its disorders through a focus on the neural mechanism. Cortex, 127, 347–370. <https://doi.org/10.1016/j.cortex.2020.03.001>

Ritter, K., Dziobek, I., Preißler, S., Rüter, A., Vater, A., Fydrich, T., Lammers, C. H., Heekeren, H. R., & Roepke, S. (2011, May). Lack of empathy in patients with narcissistic personality disorder. Psychiatry Research, 187(1–2), 241–247. <https://doi.org/10.1016/j.psychres.2010.09.013>

Rogier, G., & Velotti, P. (2018, February 17). Narcissistic Implications in Gambling Disorder: The Mediating Role of Emotion Dysregulation. Journal of Gambling Studies, 34(4), 1241–1260. <https://doi.org/10.1007/s10899-018-9759-x>

Ronningstam, E. (2014). Beyond the diagnostic traits: A collaborative exploratory diagnostic process for dimensions and underpinnings of narcissistic personality disorder. Personality Disorders: Theory, Research, and Treatment, 5(4), 434–438. <https://doi.org/10.1037/per0000034>

Rosenthal, S. A., Hooley, J. M., Montoya, R. M., van der Linden, S. L., & Steshenko, Y. (2020). The Narcissistic Grandiosity Scale: A Measure to Distinguish Narcissistic Grandiosity From High Self-Esteem. Assessment, 27(3), 487–507. <https://doi.org/10.1177/1073191119858410>

Schalkwijk, F., Luyten, P., Ingenhoven, T., & Dekker, J. (2021, July 15). Narcissistic Personality Disorder: Are Psychodynamic Theories and the Alternative DSM-5 Model for Personality Disorders Finally Going to Meet? *Frontiers in Psychology, 12.* <https://doi.org/10.3389/fpsyg.2021.676733>

Schulze, L., Dziobek, I., Vater, A., Heekeren, H. R., Bajbouj, M., Renneberg, B., Heuser, I., & Roepke, S. (2013). Gray matter abnormalities in patients with narcissistic personality disorder. *Journal of Psychiatric Research*, *47*(10), 1363–1369. https://doi.org/10.1016/j.jpsychires.2013.05.017

Stanton, K., & Zimmerman, M. (2018, May). Clinician ratings of vulnerable and grandiose narcissistic features: Implications for an expanded narcissistic personality disorder diagnosis. Personality Disorders: Theory, Research, and Treatment, 9(3), 263–272. <https://doi.org/10.1037/per0000272>

Stolz, D. S., Vater, A., Schott, B. H., Roepke, S., Paulus, F. M., & Krach, S. (2021). Reduced frontal cortical tracking of conflict between self-beneficial versus prosocial motives in Narcissistic Personality Disorder. NeuroImage: Clinical, 32, 102800. <https://doi.org/10.1016/j.nicl.2021.102800>

Tanzilli, A., & Gualco, I. (2020, March). Clinician Emotional Responses and Therapeutic Alliance When Treating Adolescent Patients With Narcissistic Personality Disorder Subtypes: A Clinically Meaningful Empirical Investigation. Journal of Personality Disorders, 34(Supplement), 42–62. <https://doi.org/10.1521/pedi.2020.34.supp.42>

Vater, A., Ritter, K., Schröder-Abé, M., Schütz, A., Lammers, C. H., Bosson, J. K., & Roepke, S. (2013, March). When grandiosity and vulnerability collide: Implicit and explicit self-esteem in patients with narcissistic personality disorder. Journal of Behavior Therapy and Experimental Psychiatry, 44(1), 37–47. <https://doi.org/10.1016/j.jbtep.2012.07.001>

Weinberg, I., & Ronningstam, E. (2022, October). Narcissistic Personality Disorder: Progress in Understanding and Treatment. FOCUS, 20(4), 368–377. <https://doi.org/10.1176/appi.focus.20220052>
